# Gut microbiota composition and butyrate production in children affected by non-IgE-mediated cow’s milk allergy

**DOI:** 10.1038/s41598-018-30428-3

**Published:** 2018-08-21

**Authors:** Roberto Berni Canani, Francesca De Filippis, Rita Nocerino, Lorella Paparo, Carmen Di Scala, Linda Cosenza, Giusy Della Gatta, Antonio Calignano, Carmen De Caro, Manolo Laiola, Jack A. Gilbert, Danilo Ercolini

**Affiliations:** 1 Department of Translational Medical Science, University of Naples Federico II, Naples, Italy; 2 European Laboratory for the Investigation of Food-Induced Diseases, University of Naples Federico II, Naples, Italy; 3 CEINGE Advanced Biotechnologies, University of Naples Federico II, Naples, Italy; 4 Task Force on Microbiome Studies, University of Naples Federico II, Naples, Italy; 5 Department of Agricultural Sciences, University of Naples Federico II, Portici, Italy; 6 Department of Pharmacy, University of Naples Federico II, Naples, Italy; 7 Division of Bioscience, Argonne National Laboratory University of Chicago, Chicago, IL USA

## Abstract

Cow’s milk allergy (CMA) is one of the earliest and most common food allergy and can be elicited by both IgE- or non-IgE-mediated mechanism. We previously described dysbiosis in children with IgE-mediated CMA and the effect of dietary treatment with extensively hydrolyzed casein formula (EHCF) alone or in combination with the probiotic *Lactobacillus rhamnosus* GG (LGG). On the contrary, the gut microbiota in non-IgE-mediated CMA remains uncharacterized. In this study we evaluated gut microbiota composition and fecal butyrate levels in children affected by non-IgE-mediated CMA. We found a gut microbiota dysbiosis in non-IgE-mediated CMA, driven by an enrichment of *Bacteroides* and *Alistipes*. Comparing these results with those previously obtained in children with IgE-mediated CMA, we demonstrated overlapping signatures in the gut microbiota dysbiosis of non-IgE-mediated and IgE-mediated CMA children, characterized by a progressive increase in *Bacteroides* from healthy to IgE-mediated CMA patients. EHCF containg LGG was more strongly associated with an effect on dysbiosis and on butyrate production if compared to what observed in children treated with EHCF alone. If longitudinal cohort studies in children with CMA will confirm these results, gut microbiota dysbiosis could be a relevant target for innovative therapeutic strategies in children with non-IgE-mediated CMA.

## Introduction

Food allergy (FA) results from an abnormal immune-mediated reaction against food antigens, such as cow’s milk proteins^1,2^. Due to its early introduction, cow’s milk allergy (CMA) is one of the earliest and most common FA^3^. The immune mechanism of CMA can be IgE-mediated or non-IgE-mediated (cell mediated) and it is recognized as a first indicator of a dysregulated immune response in the pediatric age^4^. In fact, children affected by CMA in the first year of life have an increased risk to develop other atopic manifestations in their later life^5,6^, as well as other chronic immune-mediated disorders such as inflammatory bowel diseases^7^. Therefore, understanding CMA pathogenesis is important in order to effectively prevent and manage the disease and its later life consequences. The intestinal microbiota plays a critical role in the maturation and continued education of the host immune system^8^. Evidence suggests that selected bacterial species and their metabolites from healthy gut microbiota, in particular the short-chain fatty acid butyrate, may positively modulate immune tolerance mechanisms^9–15^. On the contrary, emerging data suggest that gut microbiota dysbiosis, characterized by imbalanced composition and function of the intestinal microbes, could be associated to the development of FA^16–19^. Data on gut microbiota features in FA seem still preliminary because the general small number of observations, difference in the experimental tools used, poor characterization  of the study subjects and lack of adequate matched controls^20^. We recently demonstrated that gut microbiota in IgE-mediated CMA infants shows significantly higher diversity than that of healthy controls. Bacterial families chacteristic of the healthy infant gut, such as *Bifidobacteriaceae* were significantly decreased in the IgE-mediated CMA gut^15^. Butyrate-producing bacteria were significantly enriched by dietary treatment with extensively hydrolyzed casein formula (EHCF) with the probiotic *Lactobacillus rhamnosus* GG (LGG)^15^.

In about one third to half of CMA patients a non-IgE-mediated mechanism is recognizable^21^. Gut microbiota features in children affected by non-IgE-mediated CMA are still poorly characterized. We aimed to comparatively evaluate gut microbiota composition and butyrate production in children affected by non-IgE-mediated CMA and in healthy controls. The impact of treatment with EHCF alone or in combination with LGG was also investigated, and a comparative evaluation of gut microbiota features in IgE- and non-IgE mediated CMA was also performed.

## Results

### Study subjects

During a six month study period, 52 non-IgE-mediated CMA subjects were evaluated for the study. Four were excluded because of the presence of exclusion criteria and 2 were excluded because the lack of informed consent, thus 46 patients were enrolled in the study. According to disease state and dietary treatment, the CMA patients were subdivided in three groups: Group 1 (CMA patients at diagnosis before any dietary intervention) (n = 23); Group 2 (CMA patients treated for 6 months with extensively hydrolysed casein formula, EHCF) (n = 9); Group 3 (CMA patients treated for 6 months with EHCF containing the probiotic *L. rhamnosus* GG, LGG) (n = 14).

During the same study period, consecutive healthy children, with negative clinical history for any allergic condition visiting our center because of minimal surgical procedures or vaccination program were also enrolled in the study, Group 4 (n = 23).

Main demographic and clinical features of the study subjects and p-value of paired comparisons are reported in Table 1. In particular, the age at enrolment, when stool sampling was performed, was similar among groups. All study subjects were weaned. Study subjects enrolled in Group 1 (CMA at baseline before any dietary intervention) were on standard formula at the time of enrolment. The adherence to treatment was optimal in all subjects. Dietary habits were similar among the four groups, with the exception of the type of hypoallergenic formula used for CMA treatment in subjects enrolled in Groups 2 and 3. The hypoallergenic formula was previously prescribed by physicians when CMA diagnosis was confirmed.

**Table 1:** Main demographic and clinical features of the study population.

	Subjects with non IgE-mediated CMA					
	At diagnosis	Treated with EHCF		Treated with EHCF + LGG		Healthy subjects
	Group 1	Group 2		Group 3		Group 4
N.	23	9		14		23
Male, n (%)	12 (52.2)	6 (66.7)		8 (57.1)		9 (39.1)
Age at enrolment, months (SD)	11.4 (7.2)	11.3 (1)		14.1 (5.8)		12.9 (7.4)
Age at diagnosis, months (SD)	11.4 (7.2)	5.3 (1)		8.1 (5.8)		—
Vaginal delivery, n (%)	11 (47.8)	6 (66.7)		5 (35.7)		10 (43.5)
Birth weight, kg (SD)	3.1 (0.3)	2.9 (0.5)		2.9 (0.5)		3.1 (0.4)
Breastfeeding for at least 1 month, n (%)	19 (82.6)	8 (88.9)		9 (64.3)		14 (60.4)
Duration of breastfeeding, months (SD)	4.1 (2.7)	2.12 (2.03)		4.55 (4.1)		3.1 (2.05)
Age at weaning, month (SD)	5 (0.8)	4.9 (0.8)		4.9 (1.2)		4.7 (1)
	***p-value***					
	**Group 1** ***vs***			**Group 2** ***vs***		**Group 3** ***vs***
	**Group 2**	**Group 3**	**Group 4**	**Group 3**	**Group 4**	**Group 4**
Male, n (%)	0.694	0.769	0.375	1.000	0.243	0.286
Age at enrolment, months (SD)	0.967	0.255	0.483	0.179	0.521	0.634
Age at diagnosis, months (SD)	0.018	0.149	—	0.179	—	—
Vaginal delivery, n (%)	0.444	0.471	0.767	0.214	0.433	0.641
Birth weight, kg (SD)	0.171	0.095	0.937	0.942	0.201	0.114
Breastfeeding for at least 1 month, n (%)	1.000	0.255	0.102	0.340	0.210	0.835
Duration of breastfeeding, months (SD)	0.079	0.733	0.246	0.150	0.309	0.260
Age at weaning, month (SD)	0.724	0.661	0.323	0.944	0.681	0.741
Familial allergy risk, n (%)	1.000	1.000	0.522	1.000	0.685	0.713

p-values of paired t-test were reported for all variables.

The median (minimun-maximum) formula intake was 480 ml (400–500 ml) in Group 2 and 465 ml (400–500 ml) in Group 3. The protein (daily intake of 1–2 gr/kg) and fat (daily intake of 2.5–6.0 gr/kg) intakes were similar into the 4 study groups. All study subjects were caucasian and were from an urban area. All subjects were single child. Information about exposure to pets and/or history of maternal/infant dietary supplements were reported. Clinical manifestations in all CMA patients enrolled in Groups 1, 2 and 3 were limited to the gastrointestinal tract.

### Gut microbiota of children with non-IgE-mediated CMA differs from that of healthy controls

**Figure 1:**
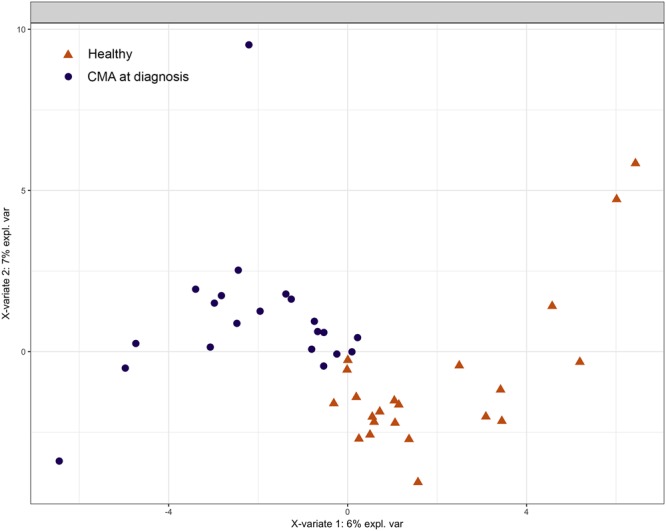
Score plot of the sPLS-DA model based on the microbiota composition at genus level of healthy and non-IgE mediated CMA subjects.

Non-IgE-mediated CMA children at diagnosis, before dietary treatment, presented significant differences in gut microbial composition when compared to healthy controls, while the alpha diversity of the microbiota was not associated with the health status (Supplementary Fig. S1). A PLS-DA model was able to discriminate healthy from non-IgE-mediated CMA subjects (Fig. 1). Only one bacterial *phylum*, Bacteroidetes, was significantly  enriched in non-IgE-mediated CMA patients (Wilcoxon pairwise tests, *p* < 0.05, Supplementary Fig. S2). However, at the level of genus, two Bacteroidetes genera, *Bacteroides* and *Alistipes*, and a single Firmicutes, *Sarcina*, were significantly enriched in non-IgE-mediated CMA when compared to healthy controls (Wilcoxon pairwise tests, *p* < 0.05, Supplementary Table S1).

**Figure 2:**
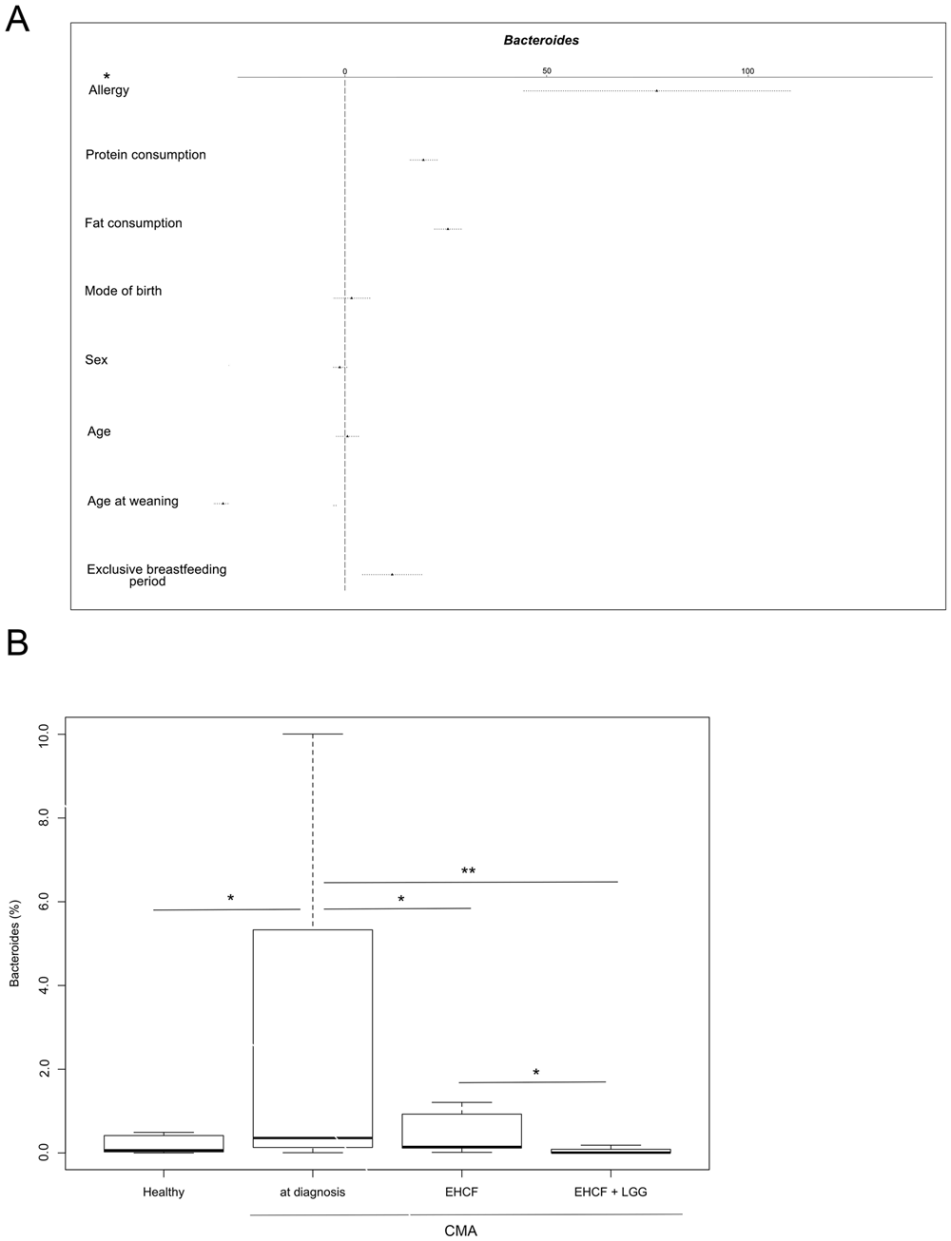
Generalized linear model fitting of patient demographic information across relative abundance of *Bacteroides* (**A**) and box plots showing the abundance of *Bacteroides* (**B**). In panel A, parallel x axis represents the relative contribution value of every factor, as predicted by the GLM model (*p < 0.05). In panel B, boxes represent the interquartile range (IQR) between the first and third quartiles, and the line inside represents the median (2nd quartile). Whiskers denote the lowest and the highest values within 1.5 x IQR from the first and third quartiles, respectively. Asterisks indicate a significant difference as obtained by pairwise Wilcoxon test (*p* < 0.05).

We applied a Generalized Linear Model (GLM) for *Bacteroides* abundance against eight features, including protein and fat consumption, mode of delivery, sex, age, age at weaning, breastfeeding duration and health status, to compare between non-IgE-mediated CMA (group 1) and healthy controls (group 4). Health status (healthy or non-IgE-mediated CMA) described the majority of the variance in the relative abundance of *Bacteroides* between these cohorts (Fig. 2A).

### Dietary management and gut microbiota composition in children with non-IgE-mediated CMA

The abundance of *Bacteroides* and *Alistipes* significantly decreased with both dietary supplementation (Supplementary Table S1) compared to initial non-IgE mediated CMA samples at diagnosis. However, the relative abundance of both *Bacteroides* and *Alistipes* was significantly lower in the samples from patients treated with EHCF + LGG (Wilcoxon pairwise tests, *p* < 0.05; Supplementary Table S1 and Fig. 2B). In addition, EHCF + LGG treated patients showed a significantly greater relative abundance of *Lachnospira*, *Ruminococcus*, *Oscillospira* compared to patients given EHCF alone (*p* < 0.05, Supplementary Table S1). Finally, *Lactobacillus* was observed at a greater relative abundance in EHCF + LGG treated children (Supplementary Fig. S3).

### Sub-genus diversity of Bacteroides differentiates healthy and non-IgE-mediated CMA subjects

**Figure 3:**
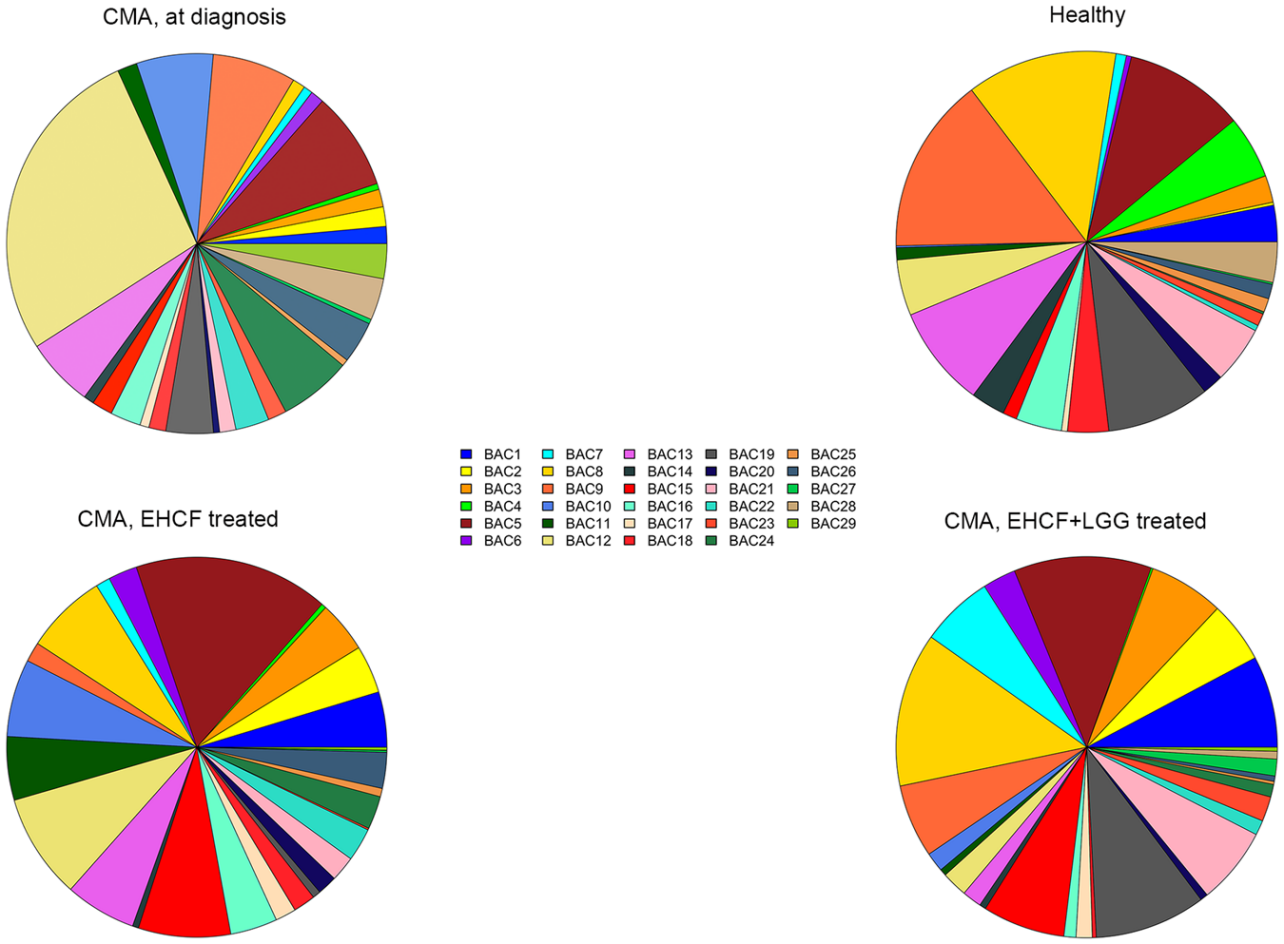
Pie charts showing the abundance of *Bacteroides* oligotypes in the different subject categories.

As *Bacteroides* had the strongest statistical association with non-IgE-mediated CMA, we further stratified the sequences annotated to this genus using oligotyping analysis. A total of 29 *Bacteroides* oligotypes were identified, and the diversity in oligotype composition was not associated to the relative abundance of the genus (Supplementary Fig. S4). CMA children maintained a greater average number of *Bacteroides* oligotypes compared to healthy subjects (11.9 vs 4.4, respectively; Wilcoxon test, *p* < 0.001), and the oligotypes that were enriched substantially differentiated healthy versus CMA children (Fig. 3). In particular, oligotypes Bac10 and Bac12 were significantly enriched and Bac8 and Bac9 were significantly reduced in CMA at diagnosis (*p* < 0.05). Both dietary interventions altered the oligotype diversity of *Bacteroides*, but EHCF + LGG resulted in a *Bacteroides* diversity pattern similar to that seen in healthy controls (Fig. 3). Indeed, the abundance of oligotypes associated with CMA (Bac10 and Bac12) was significantly reduced compared with CMA at diagnosis upon both the treatments (*p* < 0.05), but only EHCF + LGG resulted in an abundance of oligotype Bac8 similar to that found in the healthy controls (*p* > 0.05). Oligotype Bac9 also increased, but was still lower than the controls (*p* < 0.05). Oligotype representative sequences were queried against the NCBI nr database and 11 different *Bacteroides* species were identified, some showing exact match (100% identity on the whole length), with sequences in the database (Supplementary Fig. S5). Overall 11 of the oligotypes were most similar to sequences of species belonging to *B. fragilis* group (Supplementary Fig. S5).

### Dietary treatments, fecal butyrate concentration and correlation with specific gut bacteria

**Figure 4:**
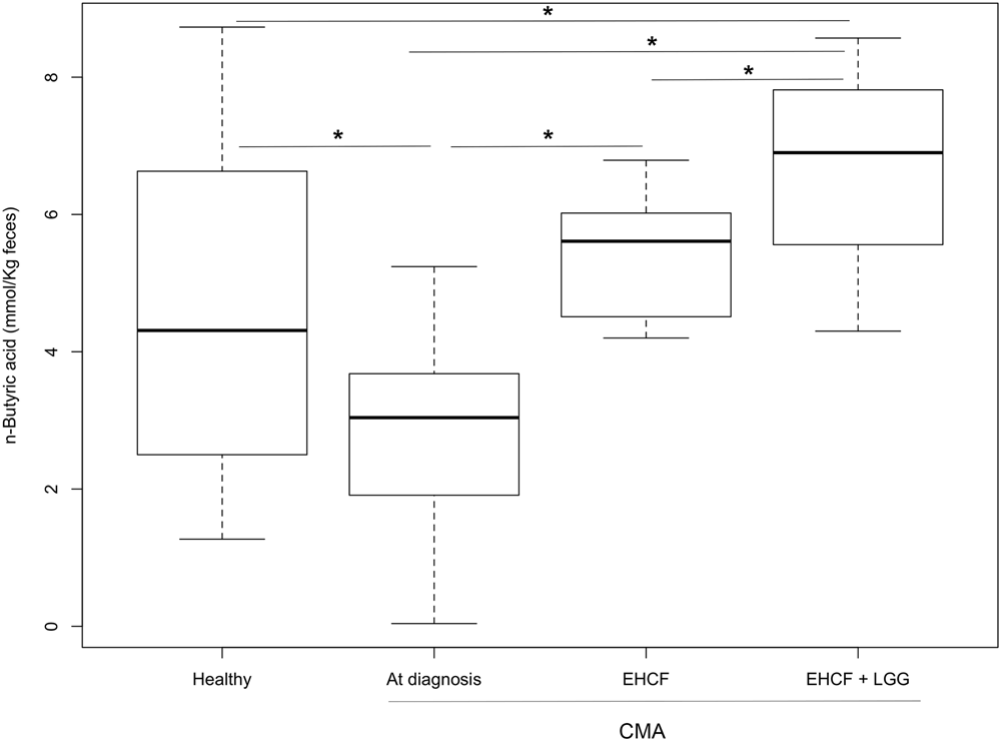
Box plots showing faecal butyrate concentration in  CMA, healthy and treated children (*p < 0.05). For a description of the box plots, see Fig. 2 legend.

Children with non-IgE-mediated CMA had a significantly lower fecal concentration of butyrate compared to healthy controls (pairwise Wilcoxon tests, *p* < 0.05). While both dietary regimens were associated to a significant increase in butyrate concentrations, the result was more evident in children treated with EHCF + LGG (Fig. 4). Butyrate concentration was significantly correlated to the relative abundance of *Lachnospira* and two *Bacteroides* oligotypes (Bac7 and Bac8) that were enriched in EHCF + LGG treated children. On the contrary, the relative abundance of oligotype Bac12, which was enriched in the CMA group, was negatively correlated to butyrate concentration.

### Gut microbiota features overlaps in IgE and non-IgE-mediated CMA children

**Figure 5:**
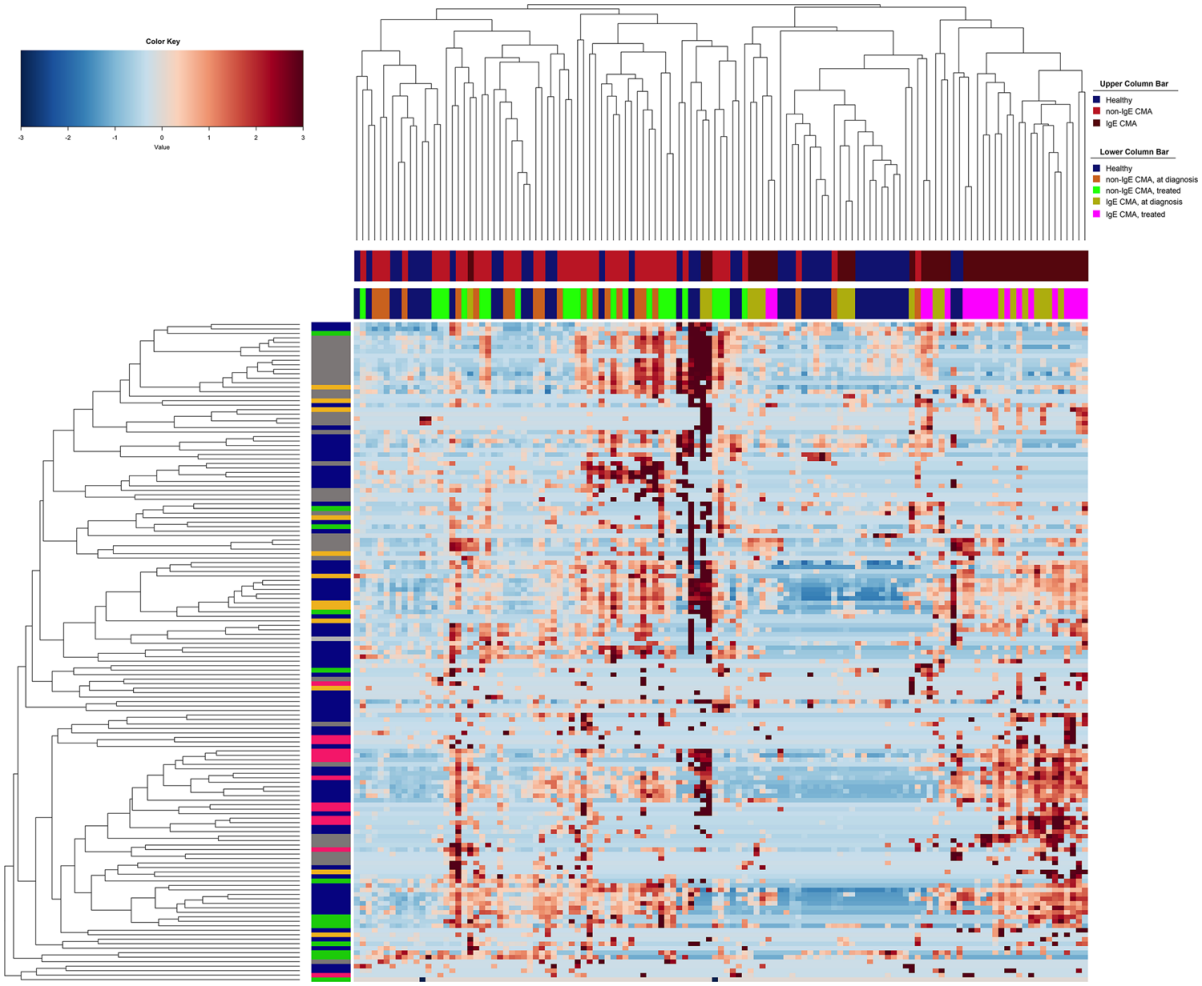
Hierarchical McQuitty-linkage clustering of the samples based on the Pearson’s correlation coefficient of the abundance of OTUs present in at least 10% of the samples. Subjects from a previously published study (14) were included. The color scale represents the scaled abundance of each variable, denoted as Z-score, with red indicating high abundance and blue indicating low abundance. Column bars are colored according to the subject categories. Row bar is colored according to the *phylum*: Actinobacteria, green; Bacteroidetes, red; Firmicutes, navy blue; Proteobacteria, grey; others, orange.

**Figure 6:**
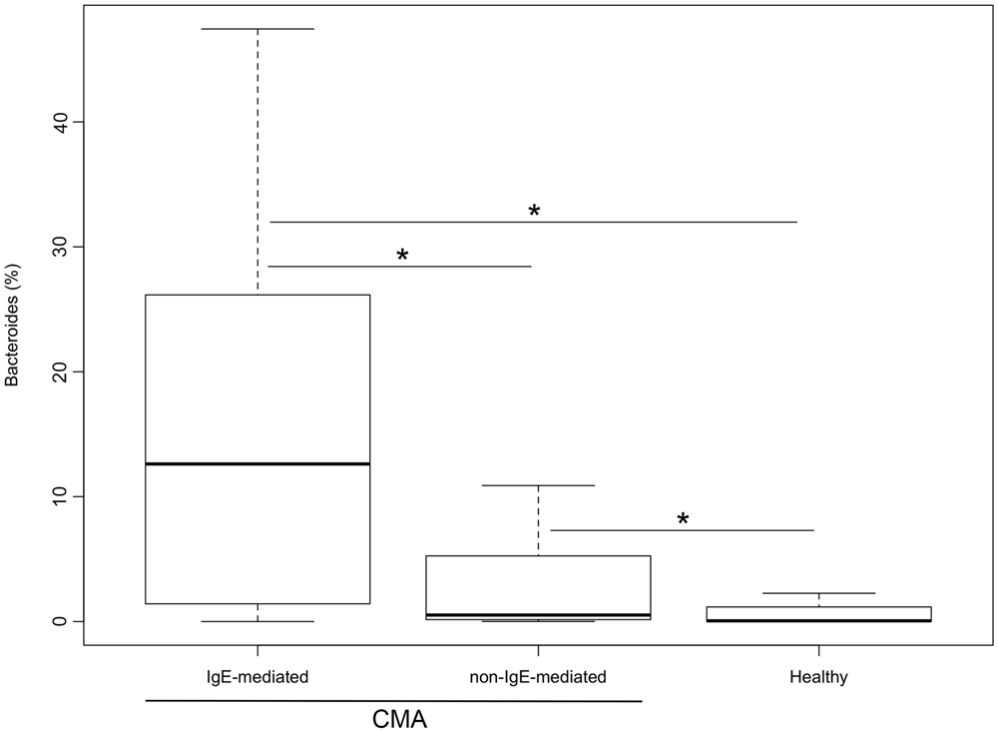
Box plots showing the abundance of *Bacteroides* in healthy, non-IgE mediated and IgE mediated CMA subjects (*p < 0.05). Subjects from a previously published study^14^ were included. For a description of the box plots, see Fig. 2 legend.

The sequence data from this study were re-analyzed alongside data produced in a previous study to compare the microbiota in non-IgE-mediated CMA *vs*. IgE-mediated CMA patients^15^. Healthy subjects from both studies clustered together in a hierarchical clustering based on Ward distance (Fig. 5). IgE-mediated CMA children at diagnosis and after treatment clearly clustered apart, indicating strong differences in gut microbiota composition, while non-IgE-CMA patients (with or without treatment) were more similar to healthy subjects (Fig. 5). This progressive gradient of dysbiosis was also clear in the PLS-DA model, where non-IgE-CMA subjects were closer to the healthy controls and separated from IgE-mediated CMA children (Supplementary Fig. S6). Accordingly, the average weighted Unifrac distance between IgE-mediated CMA and healthy subjects was significantly higher than that between non-IgE-CMA and healthy controls (0.68 ± 0.04 and 0.49 ± 0.08, respectively; *p* < 0.05). Interestingly, overlapping features characterized the gut microbiota dysbiosis in the two forms of CMA. In particular, a significant enrichment in *Bacteroides* was observed from healthy to non-IgE-mediated, and then to IgE-mediated CMA profiles (Fig. 6). *Alistipes, Fusobacterium* and *Bilophila* were significantly enriched in IgE-mediated compared to non-IgE-mediated CMA subjects (Wilcoxon test, *p* < 0.05; Supplementary Table S2), while *Eubacterium*, *Blautia*, *Akkermansia* and *Raoultella* resulted increased in non-IgE-mediated CMA patients (Supplementary Table S2).

## Discussion

We are witnessing a dramatic and apparently ongoing increase in the prevalence of FA^22^, but the cause of this increase is still largely undefined. Recent evidence has emphasized the role of intestinal bacteria in the prevention or treatment of FA, and there is mounting evidence that microbial dysbiosis early in life represents a critical factor underlying FA development^23,24^. We observed that children with non-IgE-mediated CMA had elevated relative abundances of *Bacteroides* and *Alistipes*. Different sub-genus patterns of *Bacteroides* were associated with CMA. An increase in *Bacteroides* has been associated with peanut and tree nut allergy and other atopic manifestations^25–27^, and *Bacteroides* species are reported to alter gut permeability^25,28^. Conversely, Ling and co-workers^29^ reported a decrease in Bacteroidetes in a cohort of Chinese children characterized by different types of FA. These discrepancies may be due to different variable regions of 16S rRNA gene targeted, to the low number of children evaluated in the study (non-IgE-mediated CMA children, n = 4), and to different dietary patterns or ethnicity^29^. We found that the relative abundance of *Bacteroides* was higher in children with IgE-mediated CMA compared to patients with non-IgE-mediated CMA and healthy controls, suggesting a key role of this genus in CMA pathogenesis and pointing to potential common pathways predisposing to both non-IgE- and IgE-mediated FA. Interestingly, a transition to IgE serum level positivity has been demonstrated in up to 30% of non-IgE-mediated FA subjects^21^.

Both EHCF and EHCF + LGG treatments influenced gut dysbiosis in non-IgE-mediated CMA children, but the result was more pronounced in patients treated with EHCF + LGG. Remarkably, the treatment with EHCF + LGG appeared to restore the *Bacteroides* sub-genus composition and structure, which exhibited diversity similar to that shown by the healthy controls.

Bacterial metabolites are an important communication tool between the commensal microbiota and the host immune system, and establish a broad basis for mutualism^13^. Short chain fatty acids (SCFAs) are among the most abundant microbial metabolites and play a critical role in mucosal integrity, local and systemic metabolic function and regulation of immune response^14,30–32^. In agreement with previous findings^15^, EHCF + LGG treatment signficantly increased butyrate production. This increase correlated with an enrichment of potential SCFA-producers as well as selected *Bacteroides* oligotypes. Previous clinical findings showed that dietary management with EHCF + LGG results in a higher rate of tolerance acquisition in infants with non-IgE-mediated CMA^33,34^. Our data support the hypothesis that gut microbiota dysbiosis could be a relevant target of treatment in CMA and that EHCF + LGG-based diet can be an efficient strategy for microbiome-targeted intervention.

The use of a well characterized and homogeneous study population without ethnic diversities and with similar environmental influences (all weaned and living in urban area, similar breastfeeding rate, single child, no pets and no history of maternal/infant dietary supplements) represents a major strength of this study. Conversely, the relative small number of subjects and the cross-sectional design are the major limitations. Longitudinal cohort studies in children with CMA are advocated and could better assess the development of gut microbiota during the disease course, and also in response to different therapeutic dietary strategies for CMA treatment. Moreover, although the age at enrolment (when faecal samples collection was done) was similar among the groups, we detected a significant difference in the age at diagnosis between Group 1 and 2, that might have affected the differences observed in the gut microbiota. Integrating these data with data generated throught transcriptome, epigenome, and metabolome investigations, will facilitate our understanding of FA and might drive the development of new preventive and therapeutic strategies.

## Methods

### Study subjects

From March to September 2014, 52 consecutive children (age range 1–26 months) visiting our tertiary pediatric allergy center for recent occurrence (last 2–4 weeks) of signs or symptoms of suspected non-IgE-mediated CMA, or for follow up visit after 6 months of exclusion diet upon a confirmed diagnosis of non-IgE-mediated CMA were evaluated and invited to participate in a cross sectional study. The exclusion criteria were: use of pre- or probiotic products and/or antibiotics in the previous 4 weeks; history of cow’s milk-induced anaphylaxis and/or other IgE-mediated signs of food allergy; concomitant presence of other food allergies or allergic diseases, eosinophilic disorders of the gastrointestinal tract, chronic systemic diseases, congenital cardiac defects, active tuberculosis, autoimmune diseases, immunodeficiency, chronic inflammatory bowel diseases, celiac disease, cystic fibrosis, metabolic diseases, lactose intolerance, malignancy, chronic pulmonary diseases or malformations of the gastrointestinal tract. Written informed consent was obtained from the parents/guardians of each subject. The diagnosis of non-IgE-mediated CMA was based on clinical history, negative result of skin prick test, and/or negative level of IgE serum-specific anti-cow’s milk proteins, and the results of a double blind placebo-controlled oral food challenge (DBPCFC)^33,34^. All DBPCFC were performed in a double-blind, placebo-controlled manner in the outpatient clinic on 2 separate days with a 1-week interval. Parents of patients taking antihistamines were advised to withhold these medications for 72 hours before and during the challenge. Randomization and preparation of the challenges were performed by experienced dietitians who were not directly involved in the procedures. In detail, every 20 minutes, increasing doses (0.1, 0.3, 1, 3, 10, 30, and 100 mL) of fresh pasteurized cow’s milk containing 3.5% of fat or an amino acid formula were administered. Full emergency equipment and medications (epinephrine, antihistamines, and steroids) were available. The results were assessed simultaneously by experienced pediatric allergists. Study subjects were scored for 9 items divided into 4 main categories on a 0 to 3-point scale (0, none; 1, light; 2, moderate; and 3, severe): (1) general (decreased blood pressure plus tachycardia); (2) skin (rash and urticaria/angioedema); (3) gastrointestinal (nausea or repeated vomiting, crampy-like abdominal pain, and diarrhea); and (4) respiratory (sneezing or itching, nasal congestion or rhinorrhea, and stridor deriving from upper airway obstruction or wheezing). If at least 2 of the 3 physicians independently scored one item at level 3 or 2 (or more) items at level 2, the test result was considered positive. Children were observed for up to 4 hours after the final dose and then discharged. In case of a positive DBPCFC result at any testing dose, the patient remained under observation until symptom resolution. If the patient did not show any symptoms within the first 24 hours, parents were advised to provide a single feed of 100 mL of the tested formula (verum or placebo) every day at home for 7 days. If any symptoms occurred during this period, the patients returned to the outpatient clinic on the same day. After 7 days of verum or placebo administration, the patients were examined, and the parents were interviewed at the center. Parents were asked to contact the center if any symptoms occurred in the 7 days after the DBPCFC procedures to rule out false-negative challenge results. The challenge result was considered negative if the patient tolerated the entire challenge, including the observation period. Fifty-two CMA patients were evaluated. Four patients were excluded because of the presence of exclusion criteria, and 2 were excluded for the lack of informed consent. Therefore, 46 CMA patients were included in this study. According to disease state and dietary treatment, CMA patients were divided in three groups: *group 1* included patients with non-IgE-mediated CMA at diagnosis, before any therapeutic intervention and receiving standard formula (n = 23); *group 2* (n = 9) included patients with diagnosis of non-IgE-mediated CMA after treatment for 6 months with an extensively hydrolyzed casein formula (EHCF; Nutramigen, Mead Johnson Nutrition, Evansville IN, US); *group 3* (n = 14) included patients with diagnosis of non-IgE-mediated CMA after treatment for 6 months with EHCF added with the probiotic *L. rhamnosus* GG (EHCF + LGG; Nutramigen LGG, Mead Johnson Nutrition, Evansville IN, US). The specific formula use was prescribed and adherence was checked according to the standard procedure adopted at our Center. Briefly, the parents received written instructions regarding the commercial name of the product and the formula preparation procedure. Then, the adherence to the treatment was checked monthly during the first 3 months of treatment and then every 6 months. Formula use was evaluated at each time visit by dietitians, counseling parents about issues that could arise during the elimination diet and on how to reach the daily recommended intake for Italian children. This allowed the study staff to evaluate compliance with the formula and to ensure that the patients received an appropriate quantity of formula to meet their nutritional requirements. During the same study period, consecutive healthy children (*group 4*, n = 23), with negative clinical history for any allergic condition visiting our center because of minimal surgical procedures or vaccination program were also enrolled. Anamnestic, demographic, anthropometric and clinical data were obtained from the parents of each subject and recorded in a clinical database. The 3-day dietary diary was collected from all study subjects at enrolment. All diaries were assessed using a specific software (Winfood, Medimatica srl, Colonnella, Teramo, Italy). For all study subjects, a stool sample (3 g) was collected to evaluate gut microbiota composition and fecal butyrate concentration and stored at −80 °C until analyses.

### Ethics

The study was approved by the Ethics Committee of the University of Naples Federico II and was registered in the Clinical Trials Protocol Registration System on March 14, 2014 (https://clinicaltrials.gov - ID number: NCT02087930).

All methods were performed in accordance with the relevant guidelines and regulations.

### DNA extraction and 16S sequencing

Fecal samples (about 1 g) were fully homogenized in STE buffer (100 mMNaCl, 10 mMTris-Cl pH 8.0, 1 mM EDTA pH 8.0) and centrifuged (500 × g, 1 min) in order to pellet debris. The supernatant was centrifuged again (12,000 × g, 2 min) and the pellet was used for DNA extraction with the PowerFecal DNA Isolation kit (Mo Bio Laboratories, Inc., Carlsbad, CA). V3-V4 region of the 16S rRNA gene was amplified by using primer and PCR conditions recently described^35^. PCR products were purified with the Agencourt AMPure XP beads (Beckman Coulter) and quantified using a Plate Reader AF2200 (Eppendorf). Amplicon multiplexing, pooling and sequencing were carried out following the Illumina 16S Metagenomic Sequencing Library Preparation protocol, on a MiSeq platform and using the MiSeq Reagent kit v2, leading to 2 × 250 bp, paired-end reads.

### Fecal butyrate analysis

One gram of frozen feces was diluted with saline buffer, vortexed and centrifuged (12,000 × g) for 10 min in 2 ml tubes. The supernatant was filtered (0.45 μm) and stored at −20 °C until analysis. Frozen fecal extracts were acidified with 20 μl of 85% (w/v) phosphoric acid and 0.5 ml of ethyl acetate, mixed, centrifuged (12,000 × g) for 1 h, and extracted in duplicate. About 0.5 ml of the pooled extract containing the acidified butyrate was transferred into a 2 ml glass vial and loaded onto an Agilent Technologies (Santa Clara, CA, USA) 7890 gas chromatograph (GC) system with automatic loader/injector. The GC column was an Agilent J&W DB-FFAP (Agilent Technologies) of 30 m, internal diameter 0.25 mm and film thickness 0.25 μm. The GC was programmed to achieve the following run parameters: initial temperature 90 °C, hold 0.5 min, ramp of 20 °C min^−1^ up to a final temperature of 190 °C, total run time 8.0 min, gas flow 7.7 ml min^−1^ split less to maintain 3.26 p.s.i. column head pressure, septum purge 2.0 ml min^−1^. Detection was achieved using a flame ionization detector. Peaks were identified using a mixed external standard and quantified by peak height/internal standard ratio.

### Statistical and bioinformatics analysis

All data were collected in a dedicated database and analysed by a statisticianwith IBM SPSS Statistics version 19.0 for Windows (SPSS Inc, Chicago, IL). The χ^2^ test and Fisher’s exact test were used for categorical variables. The level of significance for all statistical tests was 2-sided, *P* < 0.05.

Raw sequence quality filtering and pre-processing was carried out as recently reported^35^. Briefly, demultiplexed, forward and reverse reads were joined by using FLASH^36^. Joined reads were quality trimmed (Phred score < 20) and short reads (<250 bp) were discarded by using Prinseq^37^. High quality reads were then imported in QIIME^38^. OTUs were picked through *de novo* approach and uclust method and taxonomic assignment was obtained by using the RDP classifier and the Greengenes database^39^, following a pipeline previously reported^35^. In order to avoid biases deriving from different sequencing depth, OTU tables were rarefied to the lowest number of sequences per sample. Statistical analyses and visualization were carried out in R environment (https://www.r-project.org).

To discriminate the microbial profiles as a function of disease, a model based on projection on latent structures (PLS) in its discriminant (DA) version was built, based on the normalized abundance (log_10_) of the microbial genera identified. The R package *mixOmics* was used. Permutational Multivariate Analysis of Variance (non-parametric (PER)MANOVA) based on Jaccard and Bray Curtis distance matrices was applied with 999 permutations to detect significant differences in the overall microbial community composition, by using the *adonis* function in *vegan* package. Non-parametric Kruskal-Wallis and pairwise Wilcoxon tests were carried out in order to find OTUs differentially abundant between the groups. A Generalized Linear Model (R function *glm*) was built in order to test the importance of continuous or discrete variables available for the subjects (mode of birth, age at weaning, age at sampling, sex, months of exclusive breastfeeding, average daily consumption of proteins and fat, health status – that is, healthy or CMA) on the relative abundance of bacterial genera significantly different between healthy and CMA subjects. Spearman’s pairwise correlations were computed between OTUs or oligotypes and short-chain fatty acid abundance (*corr.test* function in *psych* package). Correction of p-values for multiple testing was performed^40^. Differences in fecal butyrate levels between the groups were evaluated by non-parametric Kruskal-Wallis and pairwise Wilcoxon tests. In order to compare the gut microbiota composition in children with non-IgE (analyzed in the present study) and IgE-mediated CMA from our previous study^15^, quality filtered reads of the previous study were downloaded from MG-RAST. Since the reads from the previous study included only V4 region of the 16S rRNAgene, they were aligned to those produced in this study, that were trimmed in 5′direction to the same length. Reads from both the studies were re-analysed as described above.

### Sub-genus diversity of *Bacteroides*

Reads assigned to *Bacteroides* genus were extracted and entropy analysis and oligotyping^41^ were carried out as described previously^42^. After the initial round of oligotyping, high entropy positions were chosen (−C option): 2, 30, 94, 104, 106, 107, 109, 114, 302, 380. To minimize the impact of sequencing errors, we required an oligotype to be represented by at least 100 reads (−M option). Moreover, rare oligotypes present in less than 10 samples were discarded (−s option). These parameters led 70,142 sequences left in the dataset. BLASTn was used to query the representative sequences against the NCBI nr database, and the top hit was considered for taxonomic assignment. Statistical analyses and visualization were carried out in R environment as described above.

### Data availability

The 16S rRNA gene sequences produced in this study are available at the Sequence Read Archive (SRA) of the National Center for Biotechnology Information (NCBI), under accession number SRP092171.

## Electronic supplementary material

Supplementary information
