# Supplementary material for: Gut microbiota composition and butyrate production in children affected by non-IgE-mediated cow’s milk allergy
Source: Sci Rep. 2018 Aug 21;8:12500. doi: 10.1038/s41598-018-30428-3 (PMC6104073; doi:10.1038/s41598-018-30428-3)
Supplement: Supplementary file 1 — Supplementary information [file 41598_2018_30428_MOESM1_ESM.pdf]

Supplementary Material

Gut microbiota composition and butyrate production in children affected by non-IgE-mediated cow's milk allergy

Roberto Berni Canani, Francesca De Filippis, Rita Nocerino, Lorella Paparo, Carmen Di Scala, Linda Cosenza, Giusy Della Gatta, Antonio Calignano, Carmen De Caro, Manolo Laiola, Jack A. Gilbert, Danilo Ercolini

Table S1. Comparative relative abundance (% of total reads) for the genera identified as significantly different between CMA non IgE patients at diagnosis and healthy controls and between the treatment groups.

**Taxa**	**Healthy subjects**	**non-IgE-mediated CMA at diagnosis**	**p value**
Sarcina	0.00	0.54	0.0044
Bacteroides	0.97	4.70	0.0270
Alistipes	0.09	0.80	0.0047
	**non-IgE-mediated CMA treated with EHCF**	**non-IgE-mediated CMA at diagnosis**	
Alistipes	0.04	0.80	0.0002
Bacteroides	0.64	4.70	0.0037
	**non-IgE-mediated CMA treated with EHCF + LGG**	**non-IgE-mediated CMA at diagnosis**	
Bacteroides	0.13	4.70	0.0096
Alistipes	0.02	0.80	0.0011
Streptomyces	0.02	0.00	0.0009
	**non-IgE-mediated CMA treated with EHCF**	**non-IgE-mediated CMA treated with EHCF + LGG**	
Bacteroides	0.64	0.13	0.0294
Alistipes	0.04	0.02	0.0441
Oscillospira	0.00	0.05	0.0297
Lachnospira	0.00	0.17	0.0145
Ruminococcus (Ruminococcaceae)	0.10	0.57	0.0410
Dialister	1.29	0.02	0.0417
Lactobacillus	0.02	0.60	0.0112

Table S2. Comparative relative abundance (% of total reads) for the genera identified as significantly different between IgE and non IgE-mediated allergies.

**Taxa**	**IgE-mediated CMA at diagnosis**	**non-IgE-mediated CMA at diagnosis**	**p value**
Bacteroides	16.99	4.70	0.0098
Alistipes	2.84	0.80	0.0378
Fusobacterium	0.02	0.00	0.0431
Bilophila	0.07	0.00	0.0235
Akkermansia	2.15	4.04	0.0019
Raoultella	0.00	1.06	0.0055
Eubacterium	0.10	0.44	0.0052
Blautia	3.93	12.70	0.0011

Supplementary Figures

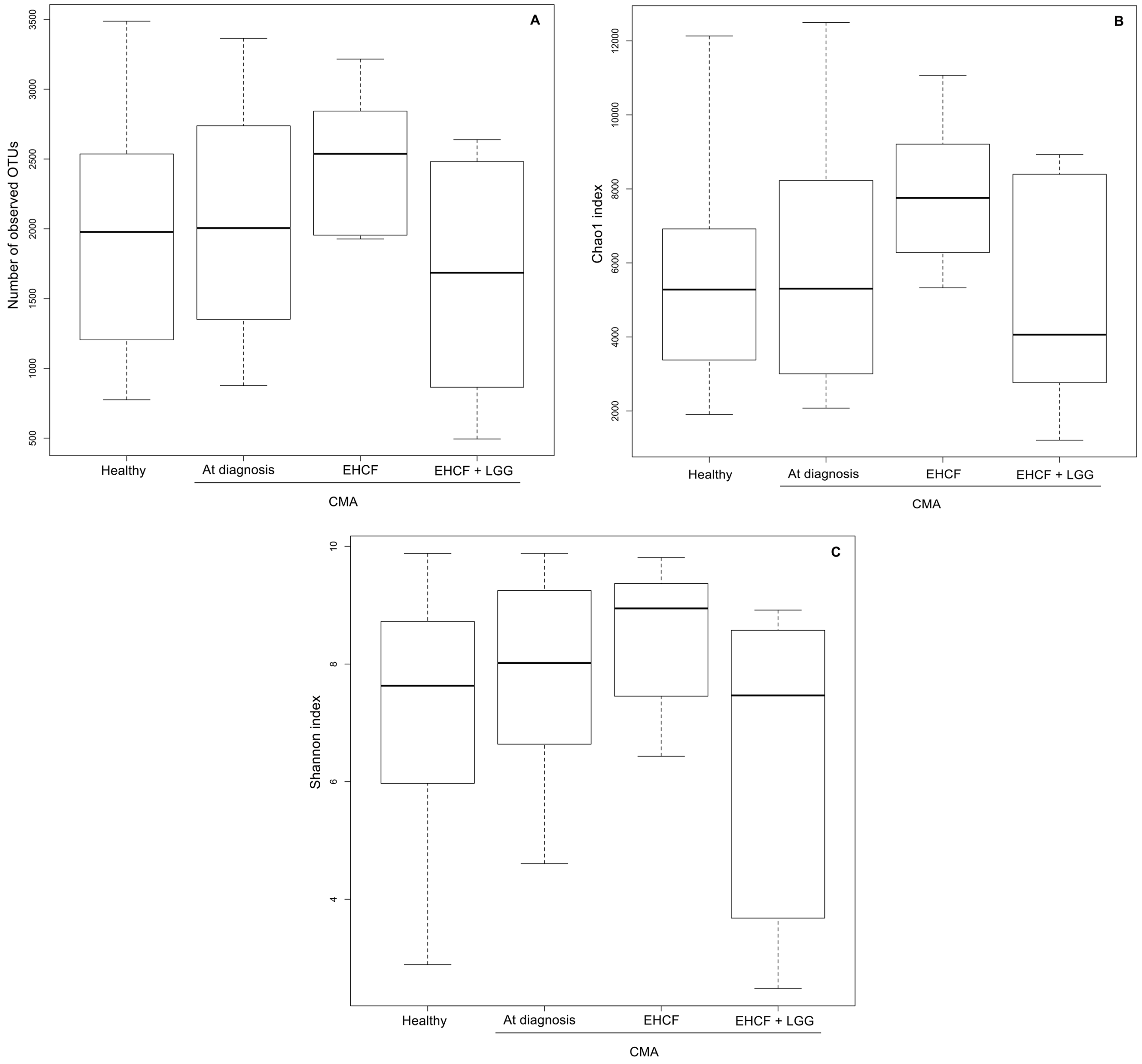

Figure S1. Box plots showing number of observed OTUs (A), Chao1 (B) and Shannon diversity (C) indices. For a description of the box plots, see Figure 2 legend.

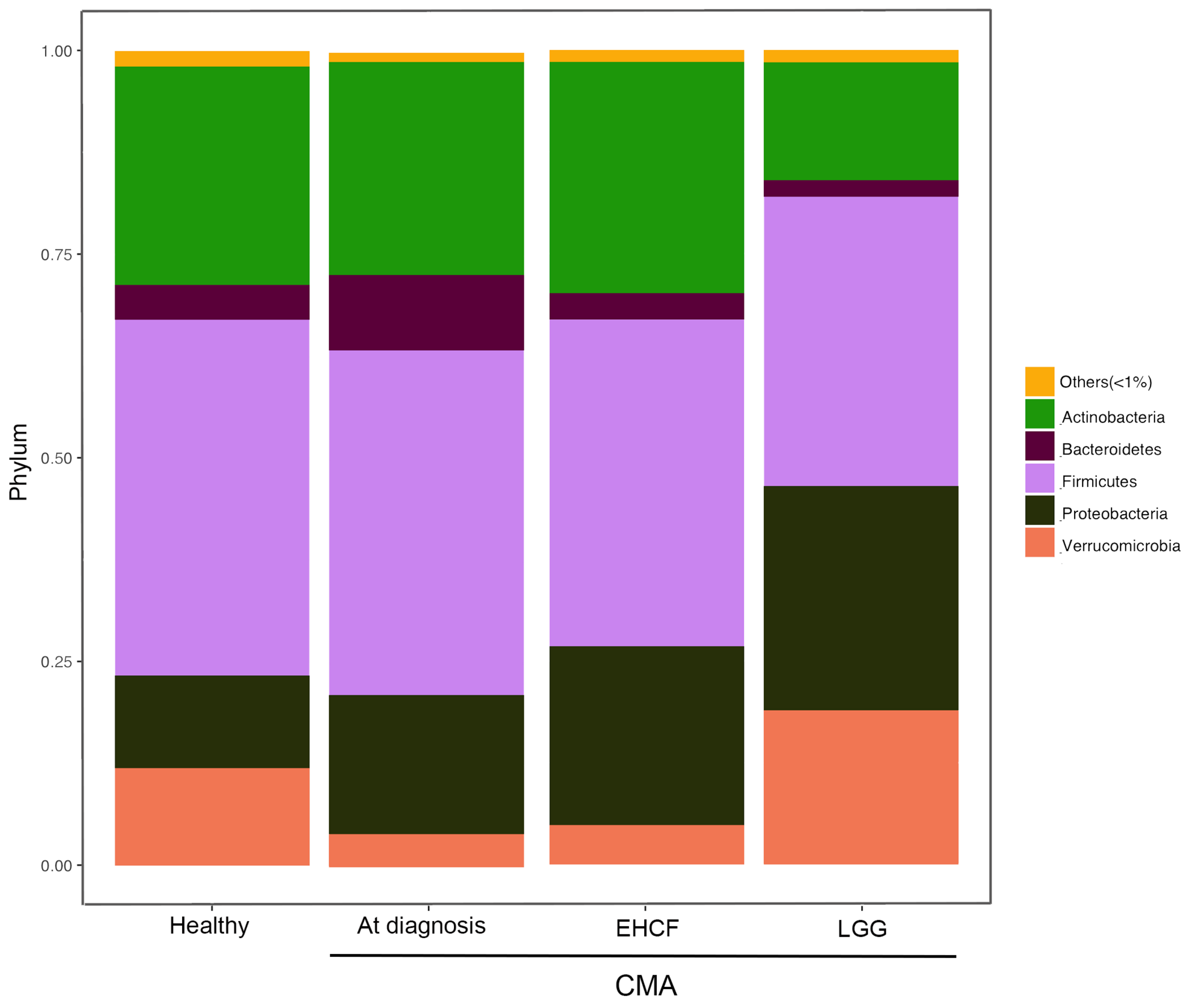

Figure S2. Stacked bar chart showing the bacterial populations at *phylum* level in the different subject categories. The average value for each group is reported.

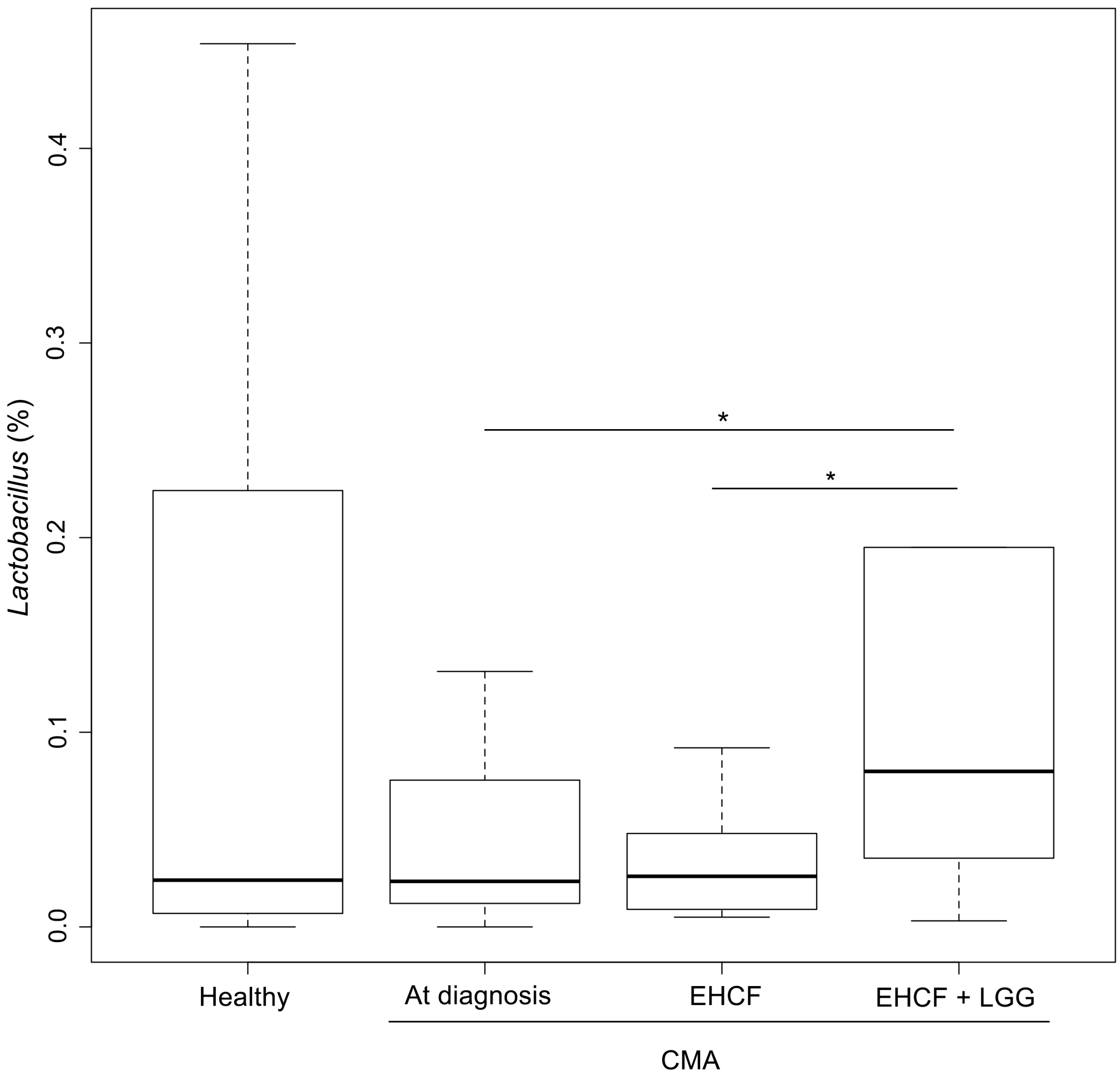

Figure S3. Box plots showing the abundance of *Lactobacillus* in the different subject categories. For a description of the box plots, see Figure 2 legend.

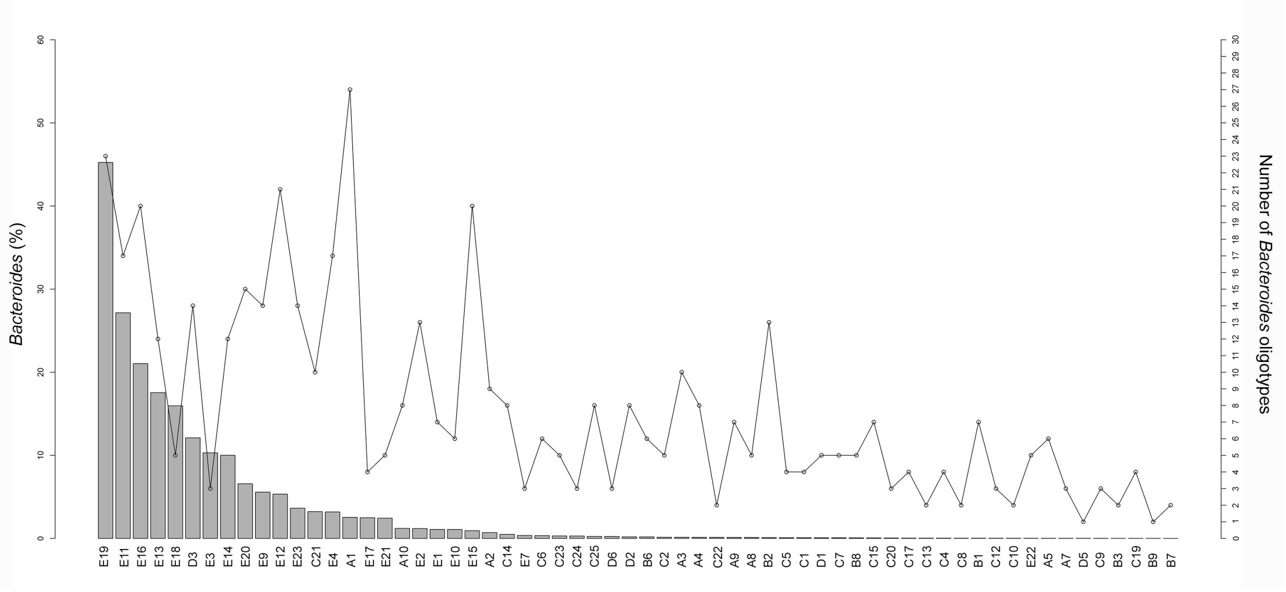

Figure S4. Bar plot of *Bacteroides* genera relative abundance ordered by size and line chart showing the number of different oligotypes identified in the same subject.

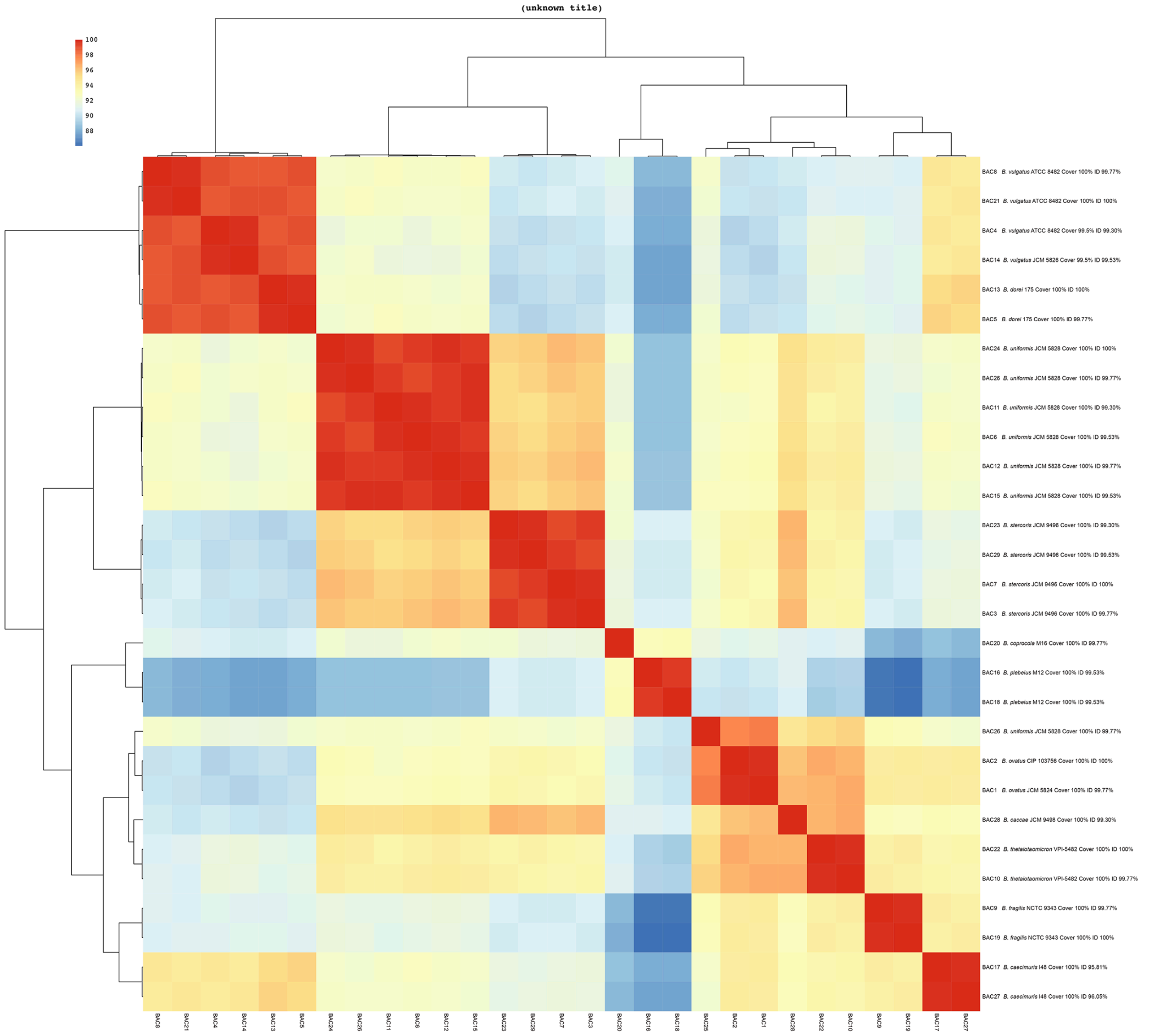

Figure S5. Heatplot showing the percent nucleotide identity between each pair of oligotypes within *Bacteroides* genus. Each oligotype is identified with the best match found in NCBI nr database, with the percent of query coverage and the percent of identity. Row and columns are clustered by Horn distance and Ward linkage hierarchical clustering.

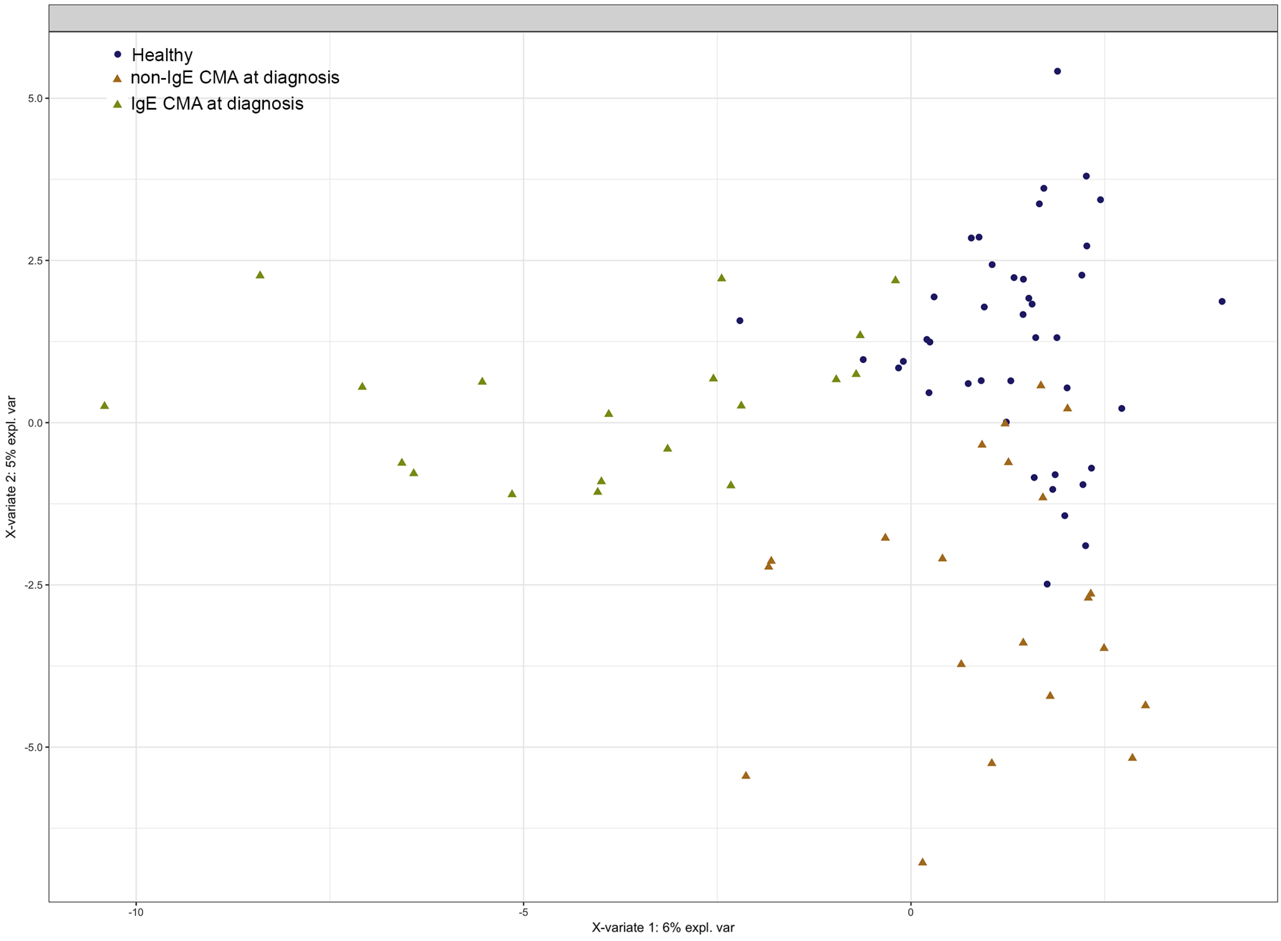

Figure S6. Score plot of the sPLS-DA model based on the microbiota composition at genus level of healthy, non-IgE mediated andIgE mediated CMA subjects. Subjects from a previously published study (14) were included.
